# Two-Dimensional Gel Electrophoresis to Study the Activity of Type IIA Topoisomerases on Plasmid Replication Intermediates

**DOI:** 10.3390/biology10111195

**Published:** 2021-11-17

**Authors:** Jorge Cebrián, Victor Martínez, Pablo Hernández, Dora B. Krimer, María-José Fernández-Nestosa, Jorge B. Schvartzman

**Affiliations:** 1 Department of Cellular and Molecular Biology, Centro de Investigaciones Biológicas (CSIC), 28040 Madrid, Spain; jorgeceb@ucm.es (J.C.); p.hernandez@cib.csic.es (P.H.); dbkrimer@cib.csic.es (D.B.K.); schvartzman@cib.csic.es (J.B.S.); 2 Department of Pharmacology and Toxicology, School of Medicine, Universidad Complutense de Madrid, Instituto de Investigación Sanitaria Gregorio Marañón, CIBERCV, 28040 Madrid, Spain; 3 Bioinformatics Laboratory, Polytechnic School, National University of Asunción, San Lorenzo P.O. Box 2111, Paraguay; vmmartinez@pol.una.py

**Keywords:** DNA topology, replication, supercoiling, pre-catenation, two-dimensional agarose gel electrophoresis

## Abstract

**Simple Summary:**

During replication, DNA molecules undergo topological changes that affect supercoiling, catenation and knotting. To better understand this process and the role of topoisomerases, the enzymes that control DNA topology in in vivo, two-dimensional agarose gel electrophoresis were used to investigate the efficiency of three type II DNA topoisomerases, the prokaryotic DNA gyrase, topoisomerase IV and the human topoisomerase 2α, on partially replicated bacterial plasmids containing replication forks stalled at specific sites. The results obtained revealed that despite the fact these DNA topoisomerases may have evolved to accomplish specific tasks, they share abilities. To our knowledge, this is the first time two-dimensional agarose gel electrophoresis have been used to examine the ability of these topoisomerases to relax supercoiling in the un-replicated region and unlink pre-catenanes in the replicated one of partially replicated molecules in vitro. The methodology described here can be used to study the role of different topoisomerases in partially replicated molecules.

**Abstract:**

DNA topoisomerases are the enzymes that regulate DNA topology in all living cells. Since the discovery and purification of ω (omega), when the first were topoisomerase identified, the function of many topoisomerases has been examined. However, their ability to relax supercoiling and unlink the pre-catenanes of partially replicated molecules has received little attention. Here, we used two-dimensional agarose gel electrophoresis to test the function of three type II DNA topoisomerases in vitro: the prokaryotic DNA gyrase, topoisomerase IV and the human topoisomerase 2α. We examined the proficiency of these topoisomerases on a partially replicated bacterial plasmid: pBR-*TerE*@AatII, with an unidirectional replicating fork, stalled when approximately half of the plasmid had been replicated in vivo. DNA was isolated from two strains of *Escherichia coli*: DH5αF’ and parE10. These experiments allowed us to assess, for the first time, the efficiency of the topoisomerases examined to resolve supercoiling and pre-catenanes in partially replicated molecules and fully replicated catenanes formed in vivo. The results obtained revealed the preferential functions and also some redundancy in the abilities of these DNA topoisomerases in vitro.

## 1. Introduction

DNA topology changes continuously as a consequence of DNA replication, transcription, recombination and chromatin remodeling [1]. To regulate these changes, several DNA topoisomerases evolved in eubacteria, archaea and eukaryotes [2].

**Figure 1:**
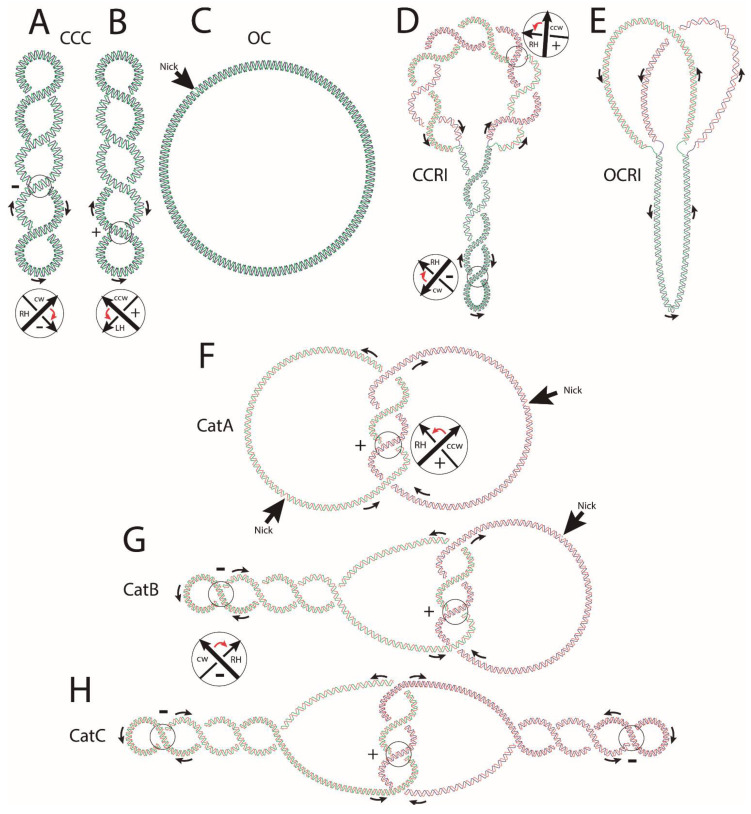
Cartoons illustrating some basic DNA topological features. (**A**) covalently closed circle (CCC) negatively supercoiled where the crossings between two oriented segments always have a negative (−) sign. (**B**) CCC positively supercoiled where the crossings between two oriented segments always have a (+) sign. The sign is determined by convention. If the direction arrow closer to the observer needs to turn clockwise to overly it with the direction arrow further from the observer, the crossing has a (−) sign. If the direction of turning is counter-clockwise, the crossing has a (+) sign. The turning angle cannot be larger than 180°. Notice that the orientations of the overlying and underlying direction arrows are not independent of each other but result from assigning a consistent direction (see the black arrows) to the DNA molecule analyzed. The DNA double-helix is shown in blue and green. (**C**) Introduction of a single “nick” (single-stranded breakage) causes the torsional energy of covalently-closed molecules to be completely released. After introducing a nick, negatively or positively supercoiled molecules gradually unwind until they reach their final, circular conformation and relaxed state named open-circle (OC). (**D**) covalently closed replication intermediate (CCRI) with un-replicated and replicated regions. Negative supercoiling in the un-replicated region facilitates the opening of the double-helix required for transcription and replication to begin and advance. This opening, however, generates (+) torsional tension ahead of the forks. At the beginning, when the unreplicated region is sufficiently large, several molecules of DNA gyrase and Topo IV acting independently from each other can eliminate all this (+) torsional tension. As replication advances and there is less space for topoisomerases to act ahead of the replication fork, (+) supercoiling transiently accumulates immediately ahead of the forks. Rotation of the forks partially releases this (+) torsional stress in the un-replicated region at the expense of generating pre-catenanes in the replicated region, where sister duplexes wind around each other with inter-duplex crossings showing (+) signs. (**E**) DNA topoisomerases gradually eliminate all this torsional tension, finally leading to an open-circle replication intermediate (OCRI). Note that in this case there are neither supercoils nor pre-catenane crossings. The parental chains are represented in blue and green, while newly synthesized chains are depicted in red. (**F**) CatA catenanes are composed of two fully replicated and relaxed rings that can be singly or multiply interlinked; (**G**) CatB catenanes are composed of one negatively supercoiled DNA molecule interlinked with another relaxed DNA ring; (**H**) CatC catenanes are composed of two negatively supercoiled and interlinked DNA rings. The parental chains are represented in blue and green, while newly synthesized chains are depicted in red. The sign of catenane crossings is (+) as newly replicated duplexes inherit the signs of pre-catenanes and the direction of the parental strands. CCC = convalently closed circle, OC = open circle, CCRI = covalently closed replication intermediate, OCRI = open-circle replication intermediate, CatAs = catenanes type A, CatBs = catenanes type B, CatCs = catenanes type C.

According to the Watson and Crick model of B-DNA, the molecule is an asymmetric right-handed (RH) double-helix [3] with major and minor grooves. These grooves are critical for the sequence-specific binding of proteins [4]. By convention, the complementary and antiparallel strands of the DNA double-helix are assigned the same direction. For this reason, all the crossings have positive (+) signs. Crossings have (+) signs when the direction of the strand above needs to turn counterclockwise to overlap the direction of the strand below. The turning angle cannot be larger than 180° [5]. In covalently-closed domains, such as DNA circles, the linking number (Lk) indicates the handedness and number of times both strands of the double-helix are linked. The DNA Lk is determined by the sum of two geometrical variables: Twist (Tw) and Writhe (Wr), according to the equation:

Lk = Tw + Wr(1)

where Tw is the number of helical turns of the DNA double-helix. Wr, on the other hand, is the number of crossings of the axis of the DNA double-helix with itself [5]. Two-dimensional agarose gel electrophoresis (2D gels) allows for the analysis of another characteristic of supercoiled DNA: ∆Lk, which is a measure of the extent of DNA supercoiling. If the ∆Lk of a given molecule is −4, for example, it indicates this molecule is underwound by 4 turns as compared to its torsional relaxed configuration [6]. Figure 1A,B represents covalently closed circles (CCCs) showing 4 negative (−) and 4 (+) supercoils with a ∆Lk nearly equal to −4 and +4, respectively. The resistance of circular DNA to deformation requires the continuity of both strands of the DNA duplex. A single nick (single-strand break) is sufficient to eliminate all torsional stress by swiveling the free ends of the broken strand around the intact one (Figure 1C). The resultant relaxed form is called an open circle (OC).

In *Escherichia coli,* DNA is negatively supercoiled [7]. During replication, progression of the replication forks demands opening of the DNA double-helix, and this process generates the formation and accumulation of (+) supercoiling ahead of the progressing forks. Two type II DNA topoisomerases, DNA gyrase and topoisomerase IV (Topo IV), cooperate to eliminate most of this (+) supercoiling, which transiently accumulates ahead of the replicating fork. However, the advancing bacterial DnaB helicase is by far more proficient at creating (+) supercoiling than DNA gyrase and Topo IV is at eliminating it [1]. It was proposed that during replication, swiveling of the progressing replication fork allows for the diffusion of some of this (+) supercoiling behind the fork, where it can be eliminated later-on [1]. In this way, the un-replicated region remains negatively supercoiled. Diffusion of each (+) supercoiling behind the fork, though, leads to the formation of two (+) intertwists of the sister duplexes for each (+) supercoil diffused from the un-replicated region (see Figure 1D). These intertwists are called “pre-catenanes” as they will become “catenanes” once replication is completed [5,6,8,9,10]. Figure 1D represents a half-replicated covalently-closed replication intermediate (CCRI) showing four (−) supercoils in the un-replicated region and eight (+) pre-catenane crossings in the replicated one. Figure 1E represents an open-circle replication intermediate (OCRI) showing no supercoiling in the un-replicated region and no pre-catenanes in the replicated one. As previously mentioned, pre-catenanes become full catenanes once replication is completed. At this time, DNA gyrase starts to introduce (−) supercoiling into one or both of the fully replicated sister duplexes [11]. This explains the formation of three types of fully replicated catenanes: catenanes type A (CatAs), formed by intertwined sister duplexes that are still nicked and therefore relaxed (Figure 1F); catenanes type B (CatBs), formed by intertwined sister duplexes where one of them is nicked and relaxed and the other covalently closed and negatively supercoiled (Figure 1G); and catenanes type C (CatCs), formed by intertwined sister duplexes where both of them are covalently closed and negatively supercoiled (Figure 1H). All these basic DNA topological conformations have been described in detail by Schvartzman et al. [6].

Type I DNA topoisomerases make transient single-stranded breaks and alter the Lk of a double-stranded DNA molecule by steps of one. Type II DNA topoisomerases, on the other hand, make transient double-stranded breaks and change the Lk by steps of two [5,12].

All known topoisomerases are arranged into five families that presumably derived from five distinct ancestral enzymes. Type I topoisomerases are grouped into three families: IA, IB and IC, whereas Type II are grouped into two families: IIA and IIB. DNA topoisomerases are named in order according to the timing of their discovery (Topo I, II, III, IV, V and VI). Type I topoisomerases have odd numbers (I, III and V) whereas type II topoisomerases have even numbers (II, IV and VI). In addition, some topoisomerases are given exclusive names, such as the ω protein for bacterial Topo IA, DNA gyrase for Topo IIA, and reverse gyrase. From an evolutionary perspective, it is widely accepted that topoisomerase I and II activities originated several times independently [2,13,14].

*Escherichia coli* cells have four topoisomerases: topoisomerase IA [15], DNA gyrase [16], topoisomerase III [17] and topoisomerase IV [18,19]. It is generally accepted that DNA gyrase is the major source of negative (−) supercoiling in vivo [20].

Four topoisomerases were also identified in eukaryotes: topoisomerase I, II, III and V. Human topoisomerase I is a type IB enzyme. It is an ATP-independent DNA single-stranded enzyme that functions during transcription and replication. In humans, there are two homologous isoforms of topoisomerase II, Topo 2α and Topo 2β. Both human type II isoforms are ATP-dependent DNA double-stranded enzymes [21].

There are conflicting reports on the essentiality of the four *E. coli* topoisomerases. DNA gyrase is unique as it is the only one able to introduce (−) supercoiling in covalently closed domains [16]. Topoisomerase IV is also called the *E. coli* decatenase. It is crucial for the segregation of fully replicated chromosomes [22,23]. It is also responsible for the unlinking of site-specific recombination products [24] and for the resolution of DNA knots [25,26]. Therefore, these two type II topoisomerases are essential in *E. coli* cells. On the contrary, these cells are apparently able to proliferate without the other two type IA topoisomerases: Topo I and Topo III. Curiously, *E. coli* cells where topoisomerase I is inhibited or even deleted are viable, but only if the cells contain compensatory mutations in DNA gyrase, RNase H or topoisomerase IV [27,28,29]. Topoisomerase III, on the other hand, which is able to remove pre-catenanes during DNA replication in vitro, is dispensable in *E. coli* cells, provided Topo IV is fully active, reflecting the specialized function of this enzyme in the unlinking of DNA replication and recombination products [29,30,31,32].

The topology of DNA replication intermediates (RIs) can be analyzed using different methods. One-dimensional and, more specifically, two-dimensional agarose gel electrophoresis allows for the simultaneous identification of thousands of molecules with different DNA topology, such as supercoiled forms, knotted forms, partially replicated forms showing pre-catenane crossings with or without reversal forks, fully replicated catenanes, and RIs containing knotted bubbles [6,9,11,23,26,33,34,35,36,37,38,39,40,41].

DNA molecules with different topologies can also be analyzed by so-called single-molecule methods, such as chromatin fiber autoradiography [42], dynamic molecular combing [43], transmission electron microscopy [38,44,45], atomic force microscopy [26,46] and magnetic tweezers [47]. Computer and numerical simulation can also be used. These methods are useful in the analysis of those DNA properties that are difficult to address experimentally [48,49,50,51]. Finally, crystallographic studies are also used to analyze the molecular structure of residues or complete topoisomerases isolated or bound to DNA [52,53,54,55]. These studies established a comprehensive picture of how topoisomerases bend and use torsion DNA to perform their function.

**Figure 2:**
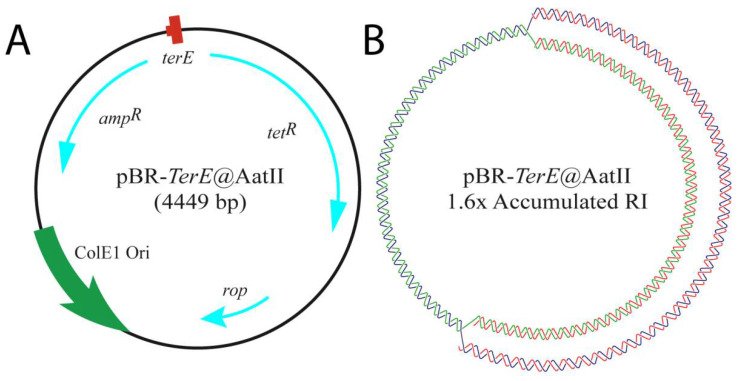
Cartoons illustrating the bacterial plasmid used in the study, pBR-*TerE@*AatII. (**A**) The genetic map at the left shows the name and mass of the plasmid. Inside, the relative positions of its most relevant features as follows: the ColE1 unidirectional origin (ColE1 Ori) in green; the rop gene and the ampicillin and tetracycline resistance genes with their relative orientations (rop, tetR and ampR) in light blue; and the *E. coli* terminator sequence (*TerE*) in red. (**B**) Cartoon showing the relaxed conformation of the accumulated replication intermediate that results when the uni-directional replicating fork stalls at *TerE*. The parental chains are represented in blue and green, while newly synthesized chains are depicted in red.

**Figure 3:**
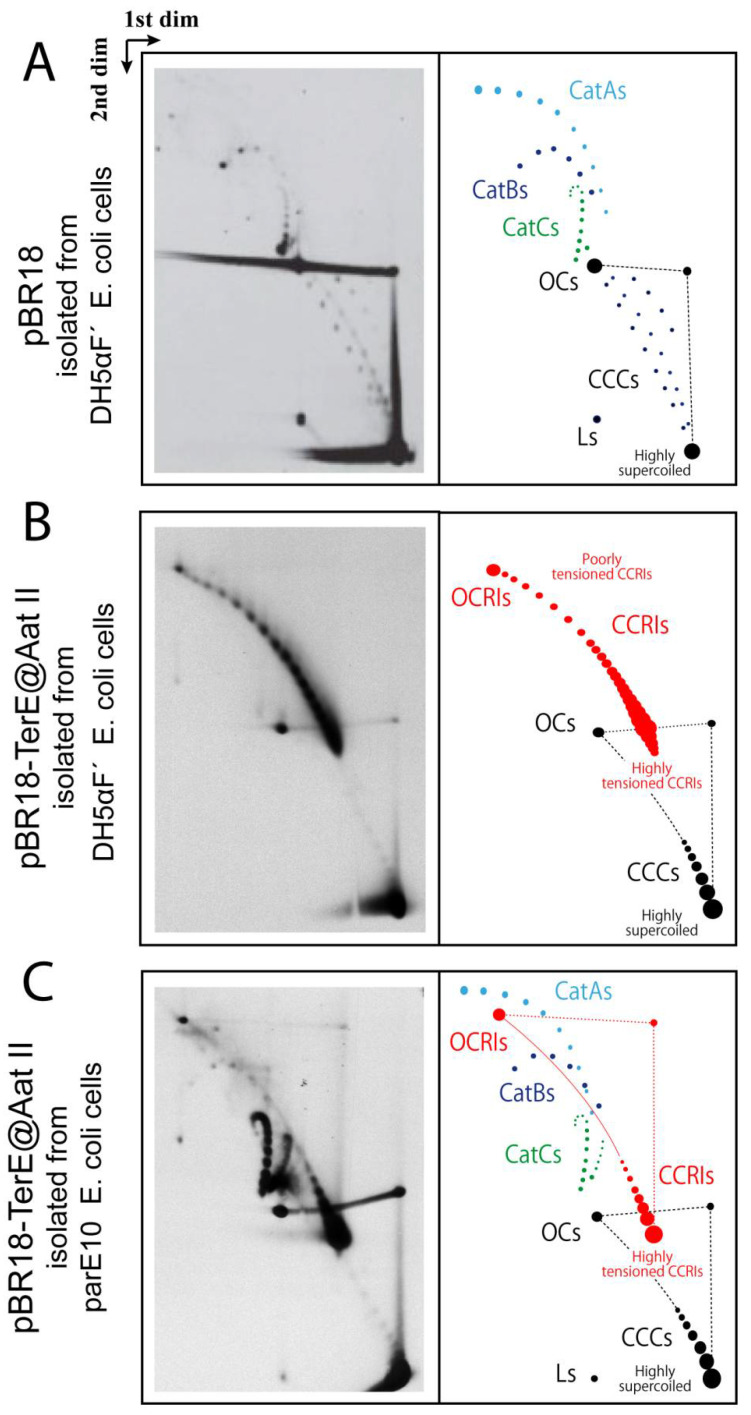
Immunograms and interpretative cartoons of intact untreated forms of pBR18 and pBR-*TerE@*AatII isolated from either DH5αF’ (**A**,**B**) or parE10 (**C**) *E. coli* cells analyzed by two-dimensional agarose gel electrophoresis. Non-replicating CCCs and OCs are depicted in black. Partially replicated CCRIs and OCRIs are depicted in red. The final position of highly supercoiled CCCs as well as highly and poorly tensioned CCRIs is indicated. In addition, when visualized, the position of CatAs is depicted in light-blue, CatBs in dark blue and CatCs in green.

In the present work, we chose to study the efficiency of three type II DNA topoisomerases in vitro: the bacterial DNA gyrase, Topo IV and human Topo 2α, on a partially replicated plasmid, pBR-*TerE*@AatII (Figure 2). This plasmid includes the 23 bp that set-up the *E. coli* chromosome replication terminator *TerE* inserted at the AatII restriction site of the plasmid [56]. The bacterial protein Tus binds *TerE,* and this complex behaves as a replication fork barrier (RFB). Blockage of the unidirectional replication fork at the Tus/*TerE* complex leads to the accumulation of partially replicated plasmids with a mass 1.6× the mass of non-replicating forms (compare Figure 3A,B). These molecules contain both supercoils in the un-replicated region and pre-catenanes in the replicated one (Figure 3B,C). We chose this plasmid because TerE is not as strong a barrier as TerB [57,58]. Not all TerE sites bind the protein Tus. For this reason, some replication forks avoid the blockage, and the plasmids are fully replicated and segregated in Topo IV proficient cells [59]. Plasmid DNA was isolated from DH5αF’ E. coli cells (Figure 3B) and from parE10 E. coli cells grown for the last 60 min at 43 °C (Figure 3C). parE codes for one of the subunits of Topo IV and the mutation leads to the accumulation of fully replicated catenanes at the restrictive temperature. We decided to use DH5αF’ instead of W3110 as wild-type because the latter is RecA^+^ and the abundance of DNA multimers makes it difficult to identify the signals of interest in 2D gels. In addition, we confirmed that the supercoiling density of pBR-*TerE*@AatII in DH5αF’ and W3110 cells are identical [60]. The proficiency of these type II DNA topoisomerases has been tested before in unreplicated and fully replicated molecules [61,62,63]. To our knowledge, though, this is the first time 2D gels have been used to analyze their efficiency on partially replicated molecules containing both supercoiling in the un-replicated region and pre-catenanes in the replicated one. The results obtained revealed the ability of these topoisomerases to relax supercoiling and unlink pre-catenanes in partially replicated molecules and fully replicated catenanes in vitro.

## 2. Materials and Methods

### 2.1. Bacterial Strains, Plasmids and Culture Medium

The *E. coli* strains used in this study were DH5αF’ {F’/gyrA96(Nalr) recA1 relA1 endA1 thi-1 hsdR17 (rk–mk+) glnV44 deoR Δ(lacZYA-argF)U169[F80dΔ(lacZ)M15]} and parE10, a derivative of W3110 {F-rph-1 IN(rrnD, rrnE)1 λ-} except [parE10 recA]. Competent cells were transformed with monomeric forms of pBR-*TerE*@AatII, a derivative of pBR322 with the polar replication terminator *TerE* [64] cloned at 60 percent of the molecules from the unidirectional ColE1 origin as described elsewhere [26,60]. Cells from overnight cultures were diluted 40-fold into fresh LB medium, grown at 37 °C to exponential phase (A600 = 0.4–0.6), quickly chilled, and centrifuged. Note that DH5αF’ cells were grown in LB medium at 37 °C while parE10 cells were grown at the restrictive temperature (43 °C) for the last 60 min [65]. In all cases, 75 µg/mL ampicillin was added to the LB medium. Isolation of plasmid DNA was performed as follows: 1000 mL of cultured cells were washed with 20 mL of STE buffer (0.1 M NaCl; 10 mM Tris-HCl, pH 8.0; and 1 mM EDTA, pH 8.0), harvested by centrifugation and resuspended in 5 mL of 25% sucrose; 0.25 M Tris-HCl, pH 8.0. Lysozyme (10 mg/mL) and RNase A (0.1 mg/mL) were added, and the suspension was maintained on ice for 5 min. Afterward, 2 mL of 0.25 M EDTA, pH 8.0 was added and the suspension was kept on ice for another 5 min. Cell lysis was achieved by adding 8 mL of lysis buffer (1% Brij-58; 0.4% sodium deoxycholate; 0.063 M EDTA, pH 8.0; and 50 mM Tris-HCl, pH 8.0) and keeping the lysate on ice for another 15 min. The lysate was centrifuged at 26,000× *g* at 4 °C for 45 min to pellet the chromosomal DNA and other bacterial debris. Plasmid DNA was recovered from the supernatant and precipitated by adding 2/3 volume of 25% polyethylene glycol 6000 and 1.25 M NaCl in TE (10 mM Tris-HCl, pH 8.0, and 1 mM EDTA) and kept overnight at 4 °C on ice. The precipitated DNA was pelleted by centrifugation at 6000× *g* at 4 °C for 15 min, and the pellet resuspended and incubated in 5 mL of a preheated digestion buffer (100 μg/mL proteinase K in 1 M NaCl, 10 mM Tris-HCl, pH 9.0, 1 mM EDTA, and 0.1% SDS), at 37 °C for 60 min. Proteins were extracted twice with phenol:chloroform:isoamyl alcohol (25:24:1) equilibrated with 10 mM Tris-HCl, pH 8.0 and then extracted once with chloroform:isoamyl alcohol (24:1). The DNA was precipitated with 2.5 volumes of absolute ethanol at −20 °C overnight and resuspended in TE.

### 2.2. DNA Treatments

*E. coli* DNA gyrase, *E. coli* Topoisomerase IV and Human Topoisomerase 2α were purchased from TopoGEN https://www.topogen.com/ (accessed on 20 October 2021). Plasmid DNA (0.2 µg/mL) was treated with different units of the topoisomerases for 30 min at 37 °C, as recommended by the manufacturer. Note that several laboratories use different concentrations of these topoisomerases with various substrates [66,67,68]. Here, we treated 0.2 μg of pBR-*TerE*@AatII plasmid DNA with different units of the topoisomerases for 30 min at 37 °C. The reactions were blocked with 100 µg/mL proteinase K (Roche) at 37 °C for 30 min (Supplementary Figures S1 and S2). Finally, for each topoisomerase we selected two treatments that achieved the best results without introducing too many double-stranded breaks. The composition (1×) of the buffers used with the topoisomerases was as follows: for Topo IV: 40 mM HEPES-KOH [pH 8], 100 mM potassium glutamate, 10 mM magnesium acetate, 10 mM dithiothreitol, 50 µg BSA/mL and 20 mM ATP. For DNA Gyrase, 35 mM Tris-Cl, pH 7.5; 24 mM KCl; 4 mM MgCl_2_; 2 mM dithiothreitol; 1.8 mM spermidine; 1 mM ATP; 6.5% glycerol; and 0.1 mg BSA/mL. For Topo 2α, 50 mM Tris-HCl, pH 8.0; 150 mM NaCl; 10 mM MgCl_2_; 5 mM ATP; 0.5 mM dithiothreitol; and 30 µg BSA/mL.

### 2.3. Two-Dimensional Agarose Gel Electrophoresis and Southern Transfer

The first dimension was in a 0.4% Seakem^®^LE agarose (Lonza Rockland, Inc., Rockland, ME, USA), gel in TBE buffer (89 mM Tris-Borate, 2 mM EDTA) at 0.9 V/cm at room temperature for 25 h. The second dimension took place in a 1% agarose gel in TBE buffer run perpendicular to the first dimension. The dissolved agarose was poured around the excised agarose lane from the first dimension, and electrophoresis occurred at 5 V/cm in a 4 °C cold chamber for 10 h. Southern transfer was performed by washing the gels for 15 min in 0.25 N HCl before an overnight transfer to positively charged nylon membranes (Roche, Basel, Switzerland) in 0.4 N NaOH.

### 2.4. Non-Radioactive Hybridization

DNA probes were labelled with digoxigenin using the DIG-High Prime kit (Roche, Basel, Switzerland). Positively charged nylon membranes (Roche, Basel, Switzerland) were pre-hybridized with 0.5 mg/mL sonicated and denatured salmon sperm DNA (Roche, Basel, Switzerland) in a 20 mL prehybridization solution (2× SSPE, 0.5% Blotto, 1% SDS, 10% dextran sulphate) at 65 °C for 4–6 h. Labeled DNA was added, and hybridization continued for another 12–16 h. The hybridized membranes were sequentially washed with 2× SSC (0.3 M NaCl in 30 mM Sodium Citrate) and 0.1% SDS at room temperature for 5 min twice and with 0.1× SSC and 0.1% SDS at 68 °C for 15 min twice as well. Detection was performed with an Antidigoxigenin-Alkaline Phosphatase conjugate antibody (Roche, Basel, Switzerland) and CDP-Star (Perkin Elmer, Inc., Waltham, MA, USA) according to the instructions provided by the manufacturer. The membranes were then exposed for different times to X-ray Agfa films (Agfa HealthCare, Mortsel, Belgium).

## 3. Results and Discussion

pBR-*TerE@*AatII DNA (Figure 2) was analyzed in high-resolution 2D agarose gel electrophoresis. The 2D gel electrophoretic mobility of non-replicating supercoiled CCCs and relaxed OCs forms as well as partially replicated forms CCRIs and OCRIs and catenated molecules (Cats) varies according to their compaction and allows their identification (Figure 3A–C). The different mobility of all these molecules has been extensively evaluated in 2D gel electrophoresis [5,56,60,69], and the topology of each population has been confirmed by electron microscopy and atomic force microscopy [26,56,70]. In pBR18, containing no RFB, the most prominent forms of the plasmid DNA isolated from DH5αF’ *E. coli* cells were supercoiled CCCs; OCs; and a few fully replicated CatAs, CatBs and CatCs (Figure 3A). In pBR-*TerE@*AatII, the most prominent forms of the plasmid DNA isolated from DH5αF’ *E. coli* cells (Figure 3B) were CCRIs and OCRIs. In addition, highly supercoiled CCCs and some OCs were also observed. In pBR-*TerE@*AatII DNA isolated from parE10 *E. coli* cells where the last 60 min of culture occurred at 43 °C (Figure 3C), the most prominent forms of the plasmid were fully replicated CatAs, CatBs and CatCs. In addition, highly supercoiled CCCs and some OCs were also observed. Note that all the replication intermediates here analyzed have the unidirectional replicating fork stalled at *TerE* [26,56,60]. The nicks responsible for the formation of OCs and OCRIs were probably induced during DNA isolation [71]. The conditions of 2D gels here used allow for the identification of CCCs with a ∆Lk between 0 and approximately 20. The signal pointing highly supercoiled CCCs corresponds to a mixture of molecules with different ∆Lks, including negatively as well as positively supercoiled circles [11,56,71].

As indicated in Material and Methods, DNA samples were treated with different concentrations of the topoisomerases (Supplementary Figures S1 and S2). Finally, for each topoisomerase we selected two concentrations that achieved the best results without introducing too many double-strand breaks, indicated by a smear of the signal corresponding to linear monomers.

**Figure 4:**
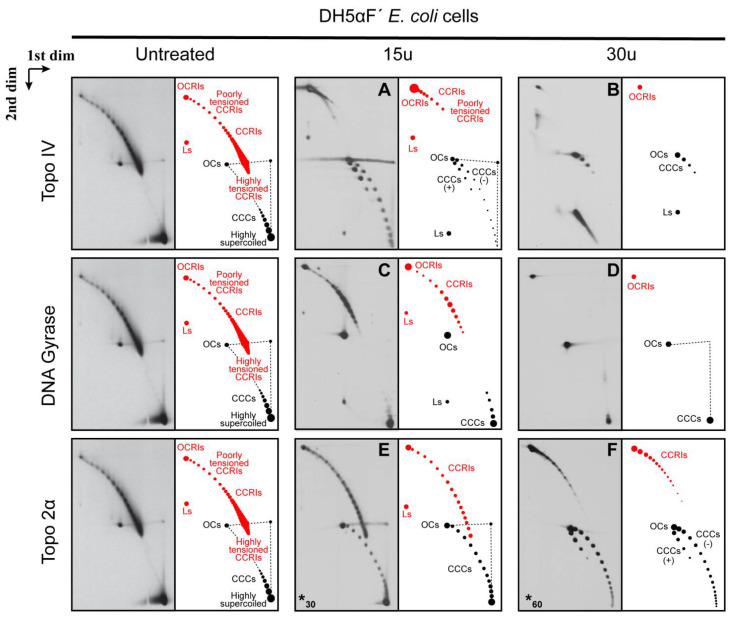
Immunograms and interpretative cartoons of intact untreated and treated forms of pBR-*TerE@*AatII isolated from DH5αF’ *E. coli* cells analyzed by two-dimensional agarose gel electrophoresis. The immunograms to the left correspond to the untreated sample and are only shown for comparison. Samples were treated with 15 units (**A**,**C**) or 30 units (**B**,**D**) of the topoisomerases in vitro, as indicated in Material and Methods, except for topo 2α, where 30 units (**E**) and 60 units (**F**) were used (indicated by an asterisk at the left down corner). Non-replicating (+) and (−) CCCs and OCs are depicted in black. Dashed straight lines correspond to smears of the most abundant species that occur during the first and second electrophoresis. CCRIs and OCRIs are depicted in red. Where it applies, fully replicated catenanes—CatAs, CatBs and CatCs—are depicted in light blue, dark blue and green, respectively. The positions of highly supercoiled as well as highly and poorly tensioned CCRIs are indicated. The immunograms on top correspond to samples treated with Topo IV; below are the immunograms that correspond to samples treated with DNA gyrase and at the bottom the immunograms that correspond to samples treated with Topo 2α as indicated in Material and Methods. * In this case a different number of units was used.

**Figure 5:**
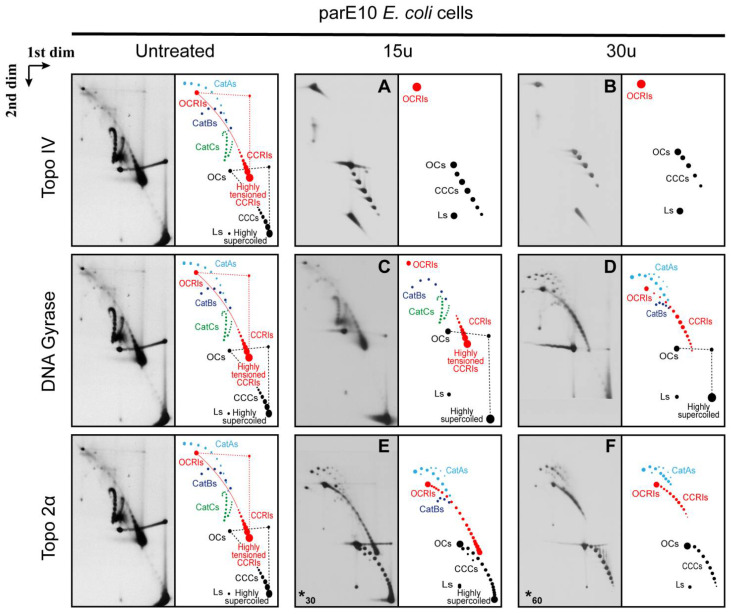
Immunograms and interpretative cartoons of intact untreated and treated forms of pBR-*TerE@*AatII isolated from parE10 *E. coli* cells where the last at 60 min occurred at the restrictive temperature (43 °C). All the samples were analyzed by two-dimensional agarose gel electrophoresis. The immunograms to the left correspond to the untreated sample and were only placed for comparison. Samples were treated with 15 units (**A**,**C**) or 30 units (**B**,**D**) of the topoisomerases in vitro, as indicated in Material and Methods, except for topo 2α, where 60 units (**E**) and 240 units (**F**) were used (indicated by an asterisk at the left down corner). CCCs and OCs are indicated in black. Dashed straight lines indicate smears of the most abundant species that occur during the first and second electrophoresis. CCRIs and OCRIs are depicted in red. Where it applies, fully replicated catenanes (CatAs, CatBs and CatCs) are depicted in light blue, dark blue and green, respectively. The position of highly supercoiled and highly tensioned CCRIs is indicated. The immunograms on top correspond to samples treated with Topo IV; below are the immunograms that correspond to samples treated with DNA gyrase and at the bottom the immunograms correspond to samples treated with Topo 2α as indicated in Material and Methods. * In this case a different number of units was used.

Topo IV relaxes the left-handed (LH) crossings of (+) supercoiling at a 20-fold faster rate than the right-handed crossings of (−) supercoiling [72]. Therefore, it should relax and probably eliminate all the highly supercoiled CCCs of the non-replicating forms (see Figure 3B). As mentioned above, Topo IV is also called the *E. coli* decatenase [22,23,26]. Therefore, it is expected to unlink and probably eliminate all the highly pre-catenated CCRIs, too (see Figure 3B). The results obtained are shown in Figure 4 and Figure 5. The immunograms corresponding to untreated samples were repeated at the left of both figures to facilitate comparison.

Treatment of the plasmid DNA isolated from DH5Af’ *E. coli* cells with 15 units of Topo IV (Figure 4A) relaxed CCCs significantly and eliminated almost all the pre-catenated CCRIs, too. Curiously, the partial relaxation of CCCs allowed for some positively supercoiled forms to be visualized. As previously mentioned, Topo IV relaxes the left-handed crossings of (+) supercoiling at a 20-fold faster rate than the right-handed crossings of (−) supercoiling, due primarily to about a 10-fold increase in processivity [72]. This observation explains how Topo IV can remove (+) supercoiling ahead of a replicating fork without relaxing all the essential (−) supercoiling of the un-replicated region in vivo [49]. Treatment of the DNA sample with 30 units of Topo IV (Figure 4B) eliminated all pre-catenated CCRIs and improved the relaxation of CCCs, too. It should be noted that the chirality of DNA may change after deproteinization in vitro. Therefore, the preferential elimination of (+) crossings by Topo IV may have a different outcome in vitro [73]. How Topo IV recognizes the chirality of DNA duplexes is still under debate, although several hypotheses have been proposed [49,74,75,76,77,78]. Moreover, results obtained in bacteria also in vitro showed that the condensin MukB interacts with Topo IV and enhances relaxation of negatively supercoiled DNA and unknotting by topoisomerase IV [62,63]. Additionally, an intense signal in the form of a sharp smear at the right bottom corner was observed with 30 units of Topo IV (Figure 4B). Judging from its electrophoretic behavior and its smear shape, our interpretation is that this signal corresponds to plasmid monomers linearized by one or more double-strand breaks. When a lower concentration of Topo IV was used (Figure 4A), this signal decreased and became a concrete dot.

DNA gyrase is the only type II DNA topoisomerase that introduces (−) supercoiling in covalently-closed domains [16,20]. Therefore, it is expected to maintain non-replicating CCCs heavily supercoiled and have little or no effect at all on pre-catenaned CCRIs.

Treatment of the DNA sample with 15 units of DNA gyrase (Figure 4C) generated an unexpected picture. There was no significant effect on the highly supercoiled CCCs. However, pre-catenated CCRIs were unlinked significantly, although not as efficiently as in the case of Topo IV. Treatment of the DNA sample with 30 units of DNA gyrase (Figure 4D) had no evident effect on CCCs but relaxed all the pre-catenated CCRIs. The contrasting activities of DNA gyrase and Topo IV have been examined in many laboratories generating conflicting results [22,24,79,80,81,82,83,84,85]. Studies using magnetic-tweezer assays showed that small changes in force and torque affect DNA gyrase activity to enhance either the introduction of (−) supercoiling, decatenation of sister duplexes or relaxation of the LH crossings of (+) supercoiling [86].

Topo 2α is the human DNA topoisomerase that relaxes (−) and (+) supercoiling and unlinks sister duplexes [5,21]. Therefore, it is expected to eliminate both highly supercoiled CCCs and pre-catenaned CCRIs.

Treatment of the DNA sample with 30 units of Topo 2α (Figure 4E) relaxed highly supercoiled molecules to some extent, and pre-catenated CCRIs were unlinked, too, although not as efficiently as in the case of a treatment with Topo IV (compare Figure 4C,E). Treatment of the DNA sample with 60 units of Topo 2α (Figure 4F) enhanced the effects observed with 30 units per µg of DNA. The decatenation efficiency improved, and the relaxation of CCCs was similar to that observed with 15 units of Topo IV (see Figure 4A). As previously mentioned, in humans Topo 2α and ß are the type II topoisomerases responsible for the relaxation of (+) and (−) supercoiling as well as for the unlinking of catenanes and pre-catenanes [5,21]. These enzymes recognize and simplify DNA topology below equilibrium values as type II DNA topoisomerases catalyze the interconversion of DNA topoisomers by transporting one DNA duplex through another [87,88]. Previous experiments using magnetic tweezers and crystallography revealed the crucial role of DNA binding and bending for these enzymes to accomplish their functions [54,55,89]. It has been shown that Topo 2α removes left-handed crossings of (+) supercoiling >10-fold faster than the right-handed crossings of (−) supercoiling [90,91]. In contrast, topoisomerase II β, which is not required for DNA replication, lacks the ability to distinguish the geometry of DNA during relaxation [91].

To summarize, we found that when pBR-*TerE@*AatII DNA was isolated from DH5αF’ *E. coli* cells, Topo IV was the most efficient enzyme in the relaxation of CCCs as well as in the unlinking CCRIs in vitro. Topo 2α showed lower relaxation efficiency than Topo IV, while DNA gyrase showed a puzzling outcome. This topoisomerase showed no significant effect on CCCs but was significantly efficient to relax pre-catenaned CCRIs. These results confirmed that in addition to its role in introducing (−) supercoiling in covalently-closed domains, DNA gyrase has unlinking activity, too [86]. Furthermore, studies performed by Ashley et al. indicated that DNA gyrase removes the left-handed crossings of (+) supercoiling at ~10-fold faster rate than it introduces right-handed crossings of (−) supercoiling into relaxed DNA [85]. Because mutations of the GyrA-box dramatically reduced its supercoiling activity [92], we hypothesize that mutations of GyrA-box that abolished the supercoiling activity could also affect the ability of DNA gyrase to resolve supercoiling and pre-catenanes in partially replicated molecules. It is also tempting to speculate that point mutations in the quinolone resistance-determining region (QRDR) of gyrA associated with a decreased DNA supercoiling [93,94] would change the efficiency of DNA gyrase to relax pre-catenaned CCRIs. These possibilities await additional investigation in future studies.

The untreated pBR-*TerE@*AatII DNA isolated from parE10 cells where the last 60 min of culture occurred at 43 °C analyzed in 2D gels generated a totally different picture (Figure 3C). Here, the presence of fully replicated CatAs, CatBs and CatCs was prominent [60]. In addition, highly supercoiled CCCs, some OCs, and CCRIs and OCRIs with the unidirectional replicating fork stalled at *TerE* were observed, too. The final conformation of CCRIs in vitro would depend on the number of crossings in the un-replicated and replicated regions, respectively, just before de-proteination. In this particular case, CCRIs isolated from parE10 cells grown for the last hour at the restrictive temperature (where TopoIV is inactive) were more torsionally tensioned than the same CCRIs isolated from DH5αF’ cells (where Topo IV is active) (see Figure 3B,C). The 2D gel electrophoretic mobility of fully replicated catenanes with different catenation numbers also varies and allows for their identification (see Figure 3C). As previously mentioned, *TerE* is not as strong a barrier as *TerB* [57,58]. This is so because its equilibrium and rate constants for binding of the Tus protein are not as high [57,58]. Hence, in a small but significant number of pBR-*TerE*@AatII molecules the unidirectional replication fork does not stall at the Tus/*TerE* complex and replication is completed. In DH5αF’ *E. coli* cells in vivo, Topo IV is active and decatenation as well as segregation occurs very fast. For this reason, fully replicated catenanes are not detected in the samples isolated from these cells (see Figure 3B). In parE10 cells grown at 43 °C, however, Topo IV is inactive [23] and the fully replicated catenanes accumulate (Figure 3C).

As stated above, Topo IV relaxes (+) as well as (−) supercoiling, although with different levels of efficiency [72]. It is also the main DNA decatenase [22,23,26]. Therefore, it is expected that in the DNA samples isolated from parE10 cells, Topo IV will relax the highly supercoiled CCCs of non-replicating forms and unlink pre-catenanes and fully replicated catenanes, too. The results obtained after treatment of the DNA sample with 15 units of Topo IV are shown in Figure 5A. Topo IV eliminated all the fully replicated catenanes (CatAs, CatBs and CatCs) and pre-catenaned CCRIs, too. It also relaxed most of the non-replicating CCCs. Treatment of the DNA sample with 30 units of Topo IV (Figure 5B) enhanced the effects observed with 15 units. In short, these treatments generated pictures similar to those observed after treatment of the DNA isolated from DH5αF’ cells (see Figure 4A,B).

DNA gyrase is essential to maintain the equilibrium level of (−) supercoiling in the *E. coli* chromosome by the wrapping-mediated mechanism. However, elevated tension on DNA favors the decatenase activity of gyrase [22] by a wrapping-independent distal T-segment capture mode [86]. Treatment of the DNA sample with 15 units of DNA gyrase (Figure 5C) was almost totally inefficient to unlink pre-catenanes as well as fully replicated catenanes. CCCs, OCs, CCRIs and OCRIs were still present, and fully replicated CatAs, CatBs and CatCs were clearly identified. Treatment of the DNA sample with 30 units of DNA gyrase (Figure 5D) generated a slightly different picture. CatCs disappeared, and the signals corresponding to CatBs and CatAs became more prominent. Notably, CCRIs were not unlinked as efficiently as in the case of DNA isolated from DH5αF’ *E. coli* cells. These results could be due to changes in the conformation of the CCRIs in the absence of Topo IV, as they would end up heavily pre-catenated in the replicated region. This could affect DNA gyrase activity in vitro to unlink sister duplexes and relax the LH crossings of (+) supercoiling [86].

Finally, as previously mentioned, Topo 2α is the human DNA topoisomerase that relaxes (−) and (+) supercoiling and unlinks sister duplexes [5,21]. Therefore, it is expected to eliminate both heavily supercoiled CCCs; pre-catenaned CCRIs; as well as fully replicated CatAs, CatBs and CatCs.

Treatment of the DNA sample with 60 units of Topo 2α (Figure 5E) generated a picture that was similar to that one generated after treatment of the DNA with 30 units of DNA gyrase (Figure 5D). CatCs disappeared, and the signals of CatAs became more prominent. The results obtained after treatment of the DNA sample with up to 240 units of Topo 2α (Figure 5F) did not change significantly except for the reduction of the signal corresponding to CCRIs. It should be noted that Topo 2α and ß evolved to deal with chromatin and not with naked DNA as in the experiments here described [2]. Moreover, TopoGEN’s specialists advise that this topoisomerase is significantly more efficient on kinetoplasts than on plasmid DNA.

To summarize, here we used for the first time 2D gels and plasmids bearing an RFB to evaluate the role of three type II DNA topoisomerases on partially replicated molecules containing both supercoiling in the un-replicated region and pre-catenanes in the replicated one. The results obtained revealed that Topo IV relaxes (+) as well as (−) supercoiling and unlinks pre-catenanes in partially replicated molecules and fully replicated catenanes with high efficiency. We found that DNA gyrase, in addition to its role in the introduction of (−) supercoiling, is also able to unlink pre-catenanes in partially replicated forms. Finally, we confirmed that Topo 2α is able to relax (+) and (−) supercoiling in non-replicating molecules and to unlink pre-catenanes in partially replicated molecules and fully replicated catenanes, although not as efficiently as Topo IV. These observations indicated that despite the fact these DNA topoisomerases may have evolved to accomplish specific tasks, they share abilities. The methodology described here can be used to study the role of different topoisomerases on partially replicated molecules in vivo using plasmids with the replication fork stalled at different sites before termination and bacterial strains with point mutations within the C-terminal domains (CTDs) of both DNA gyrase and Topo IV.

## Data Availability

The data presented in this study are available in this article and the Supplementary Materials.

## Supplementary Materials

The following are available online at https://www.mdpi.com/article/10.3390/biology10111195/s1, Figure S1: Immunograms of intact forms of pBR-TerE@AatII isolated from DH5αF’ or parE10 *E. coli* cells, treated with different concentrations of Topo IV in vitro and analyzed by two-dimensional agarose gel electrophoresis; Figure S2: Immunograms of intact forms of pBR-TerE@AatII isolated from DH5αF’ or parE10 *E. coli* cells, treated with different concentrations of Topo 2α in vitro and analyzed by two-dimensional agarose gel electrophoresis.
