# Supplementary figures and images for: Two-Dimensional Gel Electrophoresis to Study the Activity of Type IIA Topoisomerases on Plasmid Replication Intermediates
Source: Biology (Basel). 2021 Nov 17;10(11):1195. doi: 10.3390/biology10111195 (PMC8615216; doi:10.3390/biology10111195)

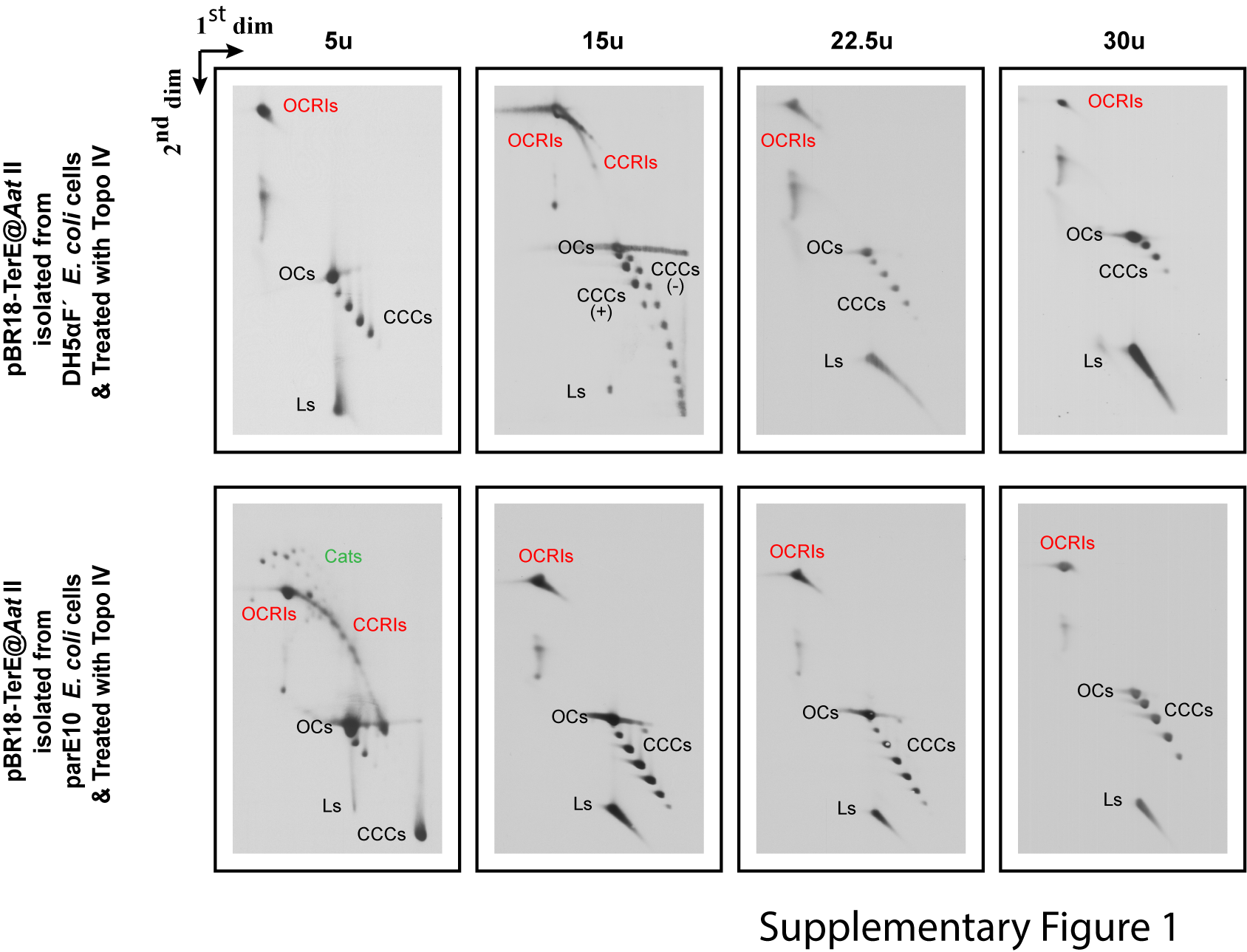

Supplement: Supplementary file 1 [file biology-10-01195-s001.zip › SupFig01.tif]

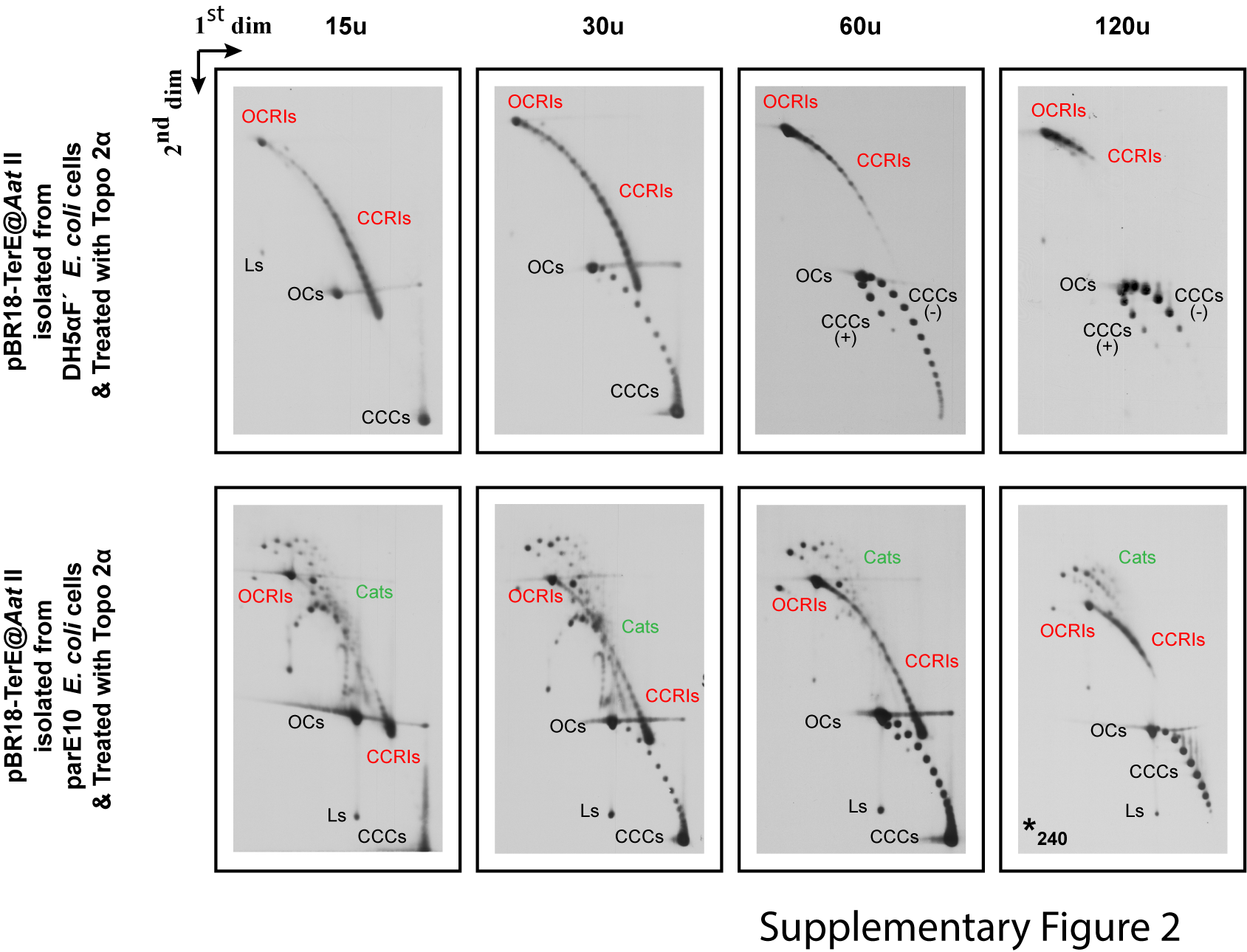

Supplement: Supplementary file 1 [file biology-10-01195-s001.zip › SupFig02.tif]
